# Bones of Contention in Institution Discipline

**Published:** 1914-10-31

**Authors:** 


### The Hospital

The Workers' Newspaper of Administrative Medicine and Institutional Life, Administration, National Insurance and Health.

No. 1480, Vol. LVII. SATURDAY, OCTOBER 31, 1914. PRICE ONE PENNY.

### BONES OF CONTENTION IN INSTITUTION DISCIPLINE.

Five-and-twenty years ago, when many of those who now constitute the most active part of the medical profession and form the staff of our hospitals were entering upon practice, there was keen competition for appointment to junior hospital and institutional posts. Such recently qualified men as were unable to obtain a house appointment in their own hospitals sought practice and experience in provincial hospitals, Poor-Law infirmaries, fever hospitals, and mental hospitals, regarding such work rather as a stepping-stone than as the beginning of a career. In such employment was often passed that stage between the light-hearted, and even rollicking student, and the sedate practitioner of orthodox bedside manners, the phases of which have often puzzled the philosopher. Salaries were low, but were in many cases equivalent to those paid to assistants in private practices, men who were willing to accept a house-surgeoncy in a teaching hospital without salary receiving £75 per annum as resident in a provincial hospital or an infirmary, or £100 per annum as a resident assistant in general practice.

With increasing demands upon those seeking medical degrees or qualifications there arose, simultaneously, an expectation of higher pay and a wider scope for the activities of the recently qualified. The increasing number of institutions and of the medical staff attached to them, the development of public health work and of the inspection of school children, the demands arising from the South African war and the sudden practical abolition of the unqualified assistant, have afforded opportunities to the young medical man previously unknown. These changes have, moreover, been concurrent with a fall in the percentage increase of registered medical men; junior posts have increased in number without a corresponding increase in the numbers of men ready and qualified to fill them. It has become increasingly difficult of recent years for such bodies as the Metropolitan Asylums Board to keep a full and efficient staff, or for men to be found to do (even temporarily) such unremunerative work as that of ship surgeon, easy and socially attractive though such work may be to the jaded student.

The young medical man finds himself to-day more sought after, commanding a higher salary, better able to select his sphere of work, and more independent than he has ever before been. Is he also better qualified for his work and ready to rise to his increased responsibilities? Judging by incidents which from time to time have been reported in The Hospital, there is reason to fear that he is not always so, and that often the institution resident expects more than his predecessors and is ready to give no more in return.

The right understanding and observance of discipline in an institution for the sick is a matter of personal character and conscience, concerning which medical education is (except by example) silent. In any such institution the members of the medical staff are not only the most responsible, but the most highly educated, and of its members is expected a high standard of regularity, tact, and discretion. Such qualities can be shown without priggishness or narrow-mindedness. It is unnecessary and out of place for the hospital resident to ape the manners of the drill-sergeant or preach the doctrines of the rabid abstainer from alcohol. There are certain points, however, in institutional discipline upon which some unselfishness is demanded, and of which there is prone to be forgetfulness. The inevitable irregularity of medical, and specially of surgical, work often serves as an excuse for needless unpunctuality; only an occasional, and very valid, excuse should account for the routine visit to wards being hurriedly made at the patients' meal-times, during visiting hours, or to the disturbance of religious services. The essential primary importance of medical work can be kept in view without causing frequent or needless delays in keeping appointments, and in the set times for the meals of nurses and of the doctor himself, for nurses' classes, etc. In an institution work must, as elsewhere, be arranged, and in that arrangement the needs of such necessary subordinate parts as the dispensary, the laboratory, the *post-mortem* room, and the admission room have to be taken into consideration.

The question of what is reasonable leave of absence also forms a frequent bone of contention.

Committees of laymen have their own views as to what constitutes a day's work, and will readily decide that absence from the early afternoon until midnight upon three weekdays in each week and freedom to play tennis upon other days indicate either a want of proper occupation or a neglect of duty. Young medical men now have widely increased interests and responsibilities, and upon their acceptance and conscientious appreciation of them, which is the secret of discipline, depend the reputation and success alike of themselves, their work, and their profession.

